# Audience effects: what can they tell us about social neuroscience, theory of mind and autism?

**DOI:** 10.1007/s40167-016-0044-5

**Published:** 2016-10-13

**Authors:** Antonia F. de C. Hamilton, Frida Lind

**Affiliations:** 1 University of Surrey, Guildford, UK; 2 Institute of Cognitive Neuroscience, University College London, Alexandra House, 17 Queen Square, London, WC1N 3AZ UK

**Keywords:** Audience effect, Social facilitation, Self-focus, Reputation management, Theory of mind, Autism

## Abstract

An audience effect arises when a person’s behaviour changes because they believe someone else is watching them. Though these effects have been known about for over 110 years, the cognitive mechanisms of the audience effect and how it might vary across different populations and cultures remains unclear. In this review, we examine the hypothesis that the audience effect draws on implicit mentalising abilities. Behavioural and neuroimaging data from a number of tasks are consistent with this hypothesis. We further review data suggest that how people respond to audiences may vary over development, personality factors, cultural background and clinical diagnosis including autism and anxiety disorder. Overall, understanding and exploring the audience effect may contribute to our models of social interaction, including reputation management and mentalising.

## Introduction

An audience effect arises when a participant’s behaviour changes because they believe another person is watching them. This effect is one of the oldest studied in psychology (Triplett 1898) and was the subject of intensive study in the 60 and 70’s, with less interest since. Taking into account a modern understanding of social cognition across a wide range of populations, we suggest it is time to look again at the audience effect. In particular, we consider whether audience effects can help us understand social cognition in diverse populations, including people with autism, people with social anxiety, people of different ages and people from different cultures. We also consider different possible cognitive mechanisms underlying the audience effect, and how we can explore them.

Two central themes are explored throughout this paper. First, what is the relationship between the audience effect and mentalising? Second, how do audiences affect different people in different ways? To explore these themes, we first provide definitions of key terms and give an overview of different theories. We briefly review major findings in typical populations using different tasks and cues to the presence of an audience. A small number of neuroimaging studies have also examined audience effects and can provide some insight into cognitive mechanisms. We will then move on to discuss individual differences, across development, clinical conditions and cultures, and to neuroimaging studies. Finally, we consider different theories about the cognitive mechanisms underlying the audience effect. Together these results can tell us more about what an audience effect is and how studying this old idea may offer new insights into real-world social interactions.

## Definitions and theories

The earliest suggestion that audiences could affect behaviour came in the work of Triplett (1898), who found that bicyclists were faster when competing against each other compared to competing against the clock. Since that point, hundreds of studies have investigated how the presence of an observer can change behaviour on a variety of tasks. Here we summarise some key issues of terminology, and define the terms we will use here. We then consider the many different theories have been put forward to account for audience effects, with varying degrees of overlap between them. These include the theories based on drive or arousal, on the self, on reputation management and on communication.

An *Audience effect* is a change in behaviour caused by *being observed by another person*, *or the belief that one is being observed by another person*. This requires at least the detection of the eyes of another and some level of awareness that the other is watching, that is, awareness of the perceptual state of the other. This contrasts which *social facilitation*, which is a change in behaviour caused by the mere presence of a conspecific (who may or may not be watching or interacting with the participant). This can be seen in a wide range of species from humans to cockroaches, implying a rather unsophisticated mechanism such as arousal (Zajonc 1965). However, in humans the mere presence of another person who is not watching does not change behaviour in the way that a watching audience does (Cottrell et al. 1968). Thus, social facilitation or mere presence effects are not the same as a true audience effect. The audience effect also contrasts with *co*-*action effects*, which are the change in behaviour when two or more individuals work on the same task. This can include joint action (Sebanz et al. 2006) but could also encompass a variety of synchronisation and behavioural coordination mechanisms. Co-action requires a much higher level of coordination than an audience effect and likely draws on more elaborate mechanisms. The present paper leaves aside both social facilitation and co-action, in order to focus only on true audience effects.

A strong theoretical account of the audience effect was put forward by Zajonc, who showed that a wide range of species, including cockroaches, rats, monkeys and humans all change their behaviour in presence of conspecifics (Zajonc 1965). He explained this in terms of a Drive theory which suggests that the presence of a conspecific increases arousal, and this change in arousal can then affect performance in various ways depending on task & context. In particular, Zajonc made the strong claim that arousal makes participants perform better on easy tasks and worse on hard tasks. Evidence for this is considered in more detail in Section “The role of task” below.

A more complex, social explanation of the audience effect is provided by Self-presentation theory (Bond 1982). This theory suggests that people maintain a public-image and consider how other people evaluate their performance when an audience is present. Being seen to make errors on hard tasks would lead to a fall in self-esteem, a feeling of embarrassment and worse performance on further trials. For example, Bond (1982) found that participant, if observed, performed poorly on easy tasks if they were presented within a difficult task. It was also found that performance on complex tasks which were embedded within easy tasks did not show an audience effect. This means that task difficulty alone does not determine the audience effect; rather Bond suggested that making mistakes impairs self-esteem which in turn affects performance.

In an update on this idea, Tennie and Frith consider the mechanisms of reputation management and describe the audience effect as a possible form of reputation management (Tennie et al. 2010). Similar to the self-presentation theory of Bond, the reputation management theory focuses on the idea that people work to maintain a good reputation in the eyes of others, which requires response modulation according to who can see an action and what task is underway. An alternative theory of audience effects can be found in the work of Fridlund, who examines the impact of being watched or imagining one is watched on the production of emotions. His studies show that people smile more when they are watched or believe they are being watched (Fridlund et al. 1990, 1992), and proposes that facial emotions have a communicative function. In this model, an audience effect arises because the participant wants to communicate something (e.g., an emotion) to a real or imagined observer. Thus, this theory implies that self-construal is inherently communicative toward an implicit audience.

These three different explanations of audience effect behaviour—in terms of self-presentation, reputation management and communication—all draw on the a number of overlapping cognitive processes. In the present paper, we focus particularly on the potential role of mentalising in the audience effect. Mentalising is the process by which people consider and manipulate other people’s mental states. It is essential in managing one’s reputation, which involves consideration of what another person thinks (Tennie et al. 2010). Mentalising is also implicit in communication, where a message is sent to change another’s knowledge state (Sperber and Wilson 1986). Finally, mentalising may have a role in self-presentation where this overlaps with reputation management (Bond 1982). A core aim of the present paper is to examine if and to what extent it is valuable to understand the audience effect in terms of mentalising processes.

There are several theoretical reasons to suggest that mentalising is central to the audience effect. First, an audience effect occurs when a participant believes he or she is being observed, and thus requires consideration of the perceptual state of another person—*can she see me*? This is similar to visual perspective taking tasks in which participants must consider which objects a person can see (Samson et al. 2010). Second, a participant who is being watched may actively consider another person’s opinions—*what does she think of me*? and this evaluation of the beliefs of another is a form of mentalising. Finally, in the presence of an audience, a participant may attempt to manipulate the opinions of the observer—a form of reputation management. Thus, participants experiencing an audience effect have several reasons to engage in mentalising.

The examination of mentalising raises two important questions. First, recent work makes a critical distinction between implicit and explicit mentalising (Apperly and Butterfill 2009). Explicit metalizing does not develop before the age of 4 years, and is typically tested with verbal tasks. People can perform with high flexibility on these tasks, but performance is relatively slow and effortful. Implicit mentalising is typically studied with eye gaze and nonverbal measures, and can be found from 6 months of age (Kovacs et al. 2010). Performance on these tasks is rapid and spontaneous but may be limited to simple situations (Apperly and Butterfill 2009). Audience effects may draw on both implicit and explicit mentalising processes depending on the context, but in the majority of the cases described below, it is likely that participants may implicitly mentalise about what others can see and their opinions.

Second, it has been claimed that audience effects are present in a range of species (Zajonc 1965), but it remains unclear if mentalising is seen in non-human species. Many cross-species effects of an ‘audience’ are likely to be due to social facilitation, and would not meet the definition of a true audience effect given above. The question of whether some animals show true audience effects (Bshary and Grutter 2006) and true mentalising (Karg et al. 2015) is beyond the scope of the present paper, and we focus only on humans. Finally, to argue that audience effect tasks invoke mentalising does not rule out the possibility that changes in arousal may also occur in the presence of an audience (as suggested by Zajonc).

Overall, the aim of the present paper is to explore the relationship between mentalising and the audience effect, to see if this can advance research in social neuroscience and help us understand diversity in social behaviour. In the following sections, we review established work on audience effects in typical populations, in order to set the scene. We then consider neuroimaging studies of audience effects—though limited in number, these can provide interesting hints about cognitive mechanisms. Finally, we focus on the diversity of social cognition, considering audience effects across development, across cultures and in atypical populations. We conclude by highlighting where further work is needed in this area.

## The audience effect in typical populations

A very large number of studies over the last 110 years have examined audience effects, though not all have clearly distinguished between audience effects and social facilitation. Here we review the major findings, and consider if they make sense within the mentalising account of the audience effect. In particular, we consider different tasks which have been used to obtain and evaluate audience effects, and different cues given to participants to indicate the presence of an audience. This review sets out the groundwork for later consideration of audience effects in diverse populations.

### The role of task

Audience effects have been reported across an enormous array of tasks, from physical and mental challenges to monetary decisions and emotion production. An aim in many studies which manipulate task or task factors is to test Zajonc’s influential claim that, in the presence of an audience, people perform better on easy tasks but worse on hard tasks (Zajonc and Sales 1965). This has been explored using a variety of tasks testing mental skill [e.g., memory or anagram solving (Geen 1985) and tasks testing physical skills such as balance or sport (Strauss 2002)]. The data generally support Zajonc’s claim, with a large meta-analysis finding effects of task as predicted (Bond and Titus 1983). However, this does not rule out more elaborate accounts, as the same data can also be accounted for under a self-presentation or reputation-management account, in which people feel embarrassed after failures on hard tasks which makes later performance worse (Bond and Titus 1983).

A second way to test the role of task and Zajonc’s drive theory is to measure physiological signals of arousal during hard and easy tasks. Being observed leads to cardiovascular changes, with increases in stress response when being observed during an difficult task and decreases when being observed an easy task (Blascovich et al. 1999). In fact, direct gaze from a real person leads to changes in skin conductance response compared to conditions without direct gaze (Myllyneva and Hietanen 2015a). Changes in arousal from direct gaze may also account for performance in a memory task (Helminen et al. 2015). These studies support Zajonc’s claim that being in the presence of another person leads to change in arousal, but do not rule out a parallel role for mentalising processes.

However, there are other task effects which are not so easy to explain under Zajonc’s drive theory. The traditional focus on task difficulty in physical and intellectual tasks does not explain the range of audience effects on social tasks or economic games (Izuma 2012). For example, donations to charity typically increase when participants donate in public, and have a high need for approval (Satow 1975). Many studies show that people are more generous and prosocial when their behaviour is observed by others (Andreoni and Petrie 2004; Bereczkei et al. 2010). For example, participants contributed more money in an investment game if they believed others would see their decisions (Van Vugt and Hardy 2010). There is no clear mechanism to manipulate task difficulty in these types of game, but rather it seems that people are motivated to donate by the desire to maintain a good ‘image’ or reputation (Ariely et al. 2009).

Audience effects are also seen in the context of emotional experience and the production of emotional facial expressions (Fridlund 1991; Fridlund et al. 1990). Many studies have reported that spontaneous or unobserved emotional faces are more ambiguous than posed or communicative facial emotions (Naab and Russell 2007; Wagner et al. 1986) and that social acceptability modulates facial expressions (Wagner and Smith 1991). Even in 10 month infants, smiles are stronger when another person is present (Jones and Raag 1989). An audience may also change feelings of emotion, as being in the presence of an empathic partner increases ratings of pain in a cold-pressor task (Hurter et al. 2014). Similar findings were found in a laser-pain task, but only in participants with avoidant attachment style (Krahe et al. 2015). These studies emphasise the communicative nature of emotion, and are compatible with the idea that implicit mentalising about how one’s own emotion is seen by others might have a role in emotion-communication audience effects.

### Cues to being observed

It is however not just the task that is important in an audience effect paradigm. A key requirement of an audience effect is that the participant is observed by another person, or believes that he/she is being observed. There are many ways in which an experimenter can induce such a belief in a participant. The simplest is to put a participant in a room with another person compared to a condition without a person present, and this method has been used in many studies (e.g., Geen and Hall 1979). However, such studies do not clearly distinguish audience effects from mere-presence effects. It can also be hard to give participants the feeling that they are not observed in the control condition, if they are in an experimental setting and know that an experimenter nearby will examine their data. Thus, experimenters have used a variety of other methods to tap into the audience effect, including explicit instructions and implicit cues.

Explicit instructions can induce an audience effect by telling participants that someone else can see them in some conditions but not in others. For example, participants may be told someone is watching over a video link (Landers et al. 1978; Somerville et al. 2013) and robust audience effects can occur even though participants have no visual access to the observer. Audience effects also arise if participants are instructed to imagine or remember another person (Fridlund 1991). In contrast, if participants can see another person but believe that a semi-silvered mirror means that the other person can’t see them, they show a reduced skin conductance response to direct gaze (Myllyneva and Hietanen 2015b). Thus, explicit instructions can substantially alter the audience effect, both in the presence and absence of the visual form of a watching person.

Video images or virtual reality can give participants the feeling of being watched. In fMRI studies, where a live audience is not feasible, participants might see video of other people ‘watching from an adjacent room’, and audience effects can be seen (Izuma et al. 2010b). Other studies manipulate the presence of a video or virtual audience without any explicit instructions. For example, an image of an animated character lowered the number of accurately completed tasks when shown on the screen (Rickenberg and Reeves 2000). In two studies, participants performed a variety of computer tasks, both hard and easy, in the presence of a virtual human or without a virtual human. Results replicated the classic audience effect, with better performance on easy tasks and worse performance on hard tasks (Park and Catrambone 2007; Zanbaka et al. 2007). These studies suggest that a virtual or video audience can change performance in the same way as a live audience.

Finally, a series of studies suggest that even simpler, less explicit cues can change social behaviour. For example, a poster showing a pair of eyes lead to more prosocial behaviour (paying for drinks or tidying up) compared to a poster of flowers (Bateson et al. 2006; Ernest-Jones et al. 2011). In economic games, adding images of eyes or even stylised eyes to the screen background can lead to more sharing in a dictator game (Haley and Fessler 2005; Oda et al. 2011) and stronger in-group favouritism (Mifune et al. 2010). In such contexts, it is unlikely that participants would state that the eyes on the poster can see them if they were asked this question. Nevertheless, the eye images may be enough to tap into an implicit feeling of being watched and thus to alter behaviour in a similar manner to an audience effect. However, it remains to be seen how general and influential this implicit audience effect might be, because photos of faces do not induce the same SCR changes as live faces (Pönkänen et al. 2011). Altogether, these studies show that a variety of cues and signals can induce an audience effect. While the effect is clearest when participants are observed by a real person, both explicit instructions and implicit cues can, in the right context, tap into the same mechanisms and alter behaviour in the same way.

### Summary

The brief reviews above highlight two clear conclusions. First, audience effects are a very general phenomena which can be observed in all tasks tested—social, emotional, economic, intellectual and physical tasks. Second, a variety of cues ranging from explicit instructions, eye contact and implicit suggestions of gaze can give rise to an audience effect. These patterns of results are all coherent with a mentalising account of the audience effect, in which being seen by another person engages processes of self-presentation and reputation management. Participants would then behave in a different fashion, in order to communicate a particular impression or emotion to the observer. However, there may also be an important role for arousal in the effects described above, as different tasks and observation conditions may lead to different arousal levels (Zajonc 1965).

It is interesting to note that the contrast between mentalising accounts of the audience effect and arousal accounts has a parallel in current theories of eye contact. Several studies show that eye contact is processes via a rapid, probably sub-cortical pathway and has strong arousal effects (Senju and Johnson 2009). However, eye contact can also induce mentalising (Kampe et al. 2003) and change social behaviours such as mimicry (Wang et al. 2011). In distinguishing between arousal models of eye contact and mentalising models, it has been helpful to turn to neuroimaging data. Thus, we also examine neuroimaging studies to advance our understanding of the cognitive processes underlying the audience effect.

## Neural mechanisms of audience effects

Neuroscientific methods can provide unique insights into audience effects, by allowing the measurement of physiological and neural processes when participants are or are not being watched. It is possible to measure arousal in terms of changes in skin conductance and pupil size. fMRI brain scanning studies can show which regions are engaged when a participant feels they are being watched, and thus to make links between the cognitive processes engaged during an audience effect and those engaged during other tasks. A key question for neuroimaging studies is making a distinction between mentalising processes and arousal. Mentalising is strongly linked to medial prefrontal cortex (mPFC) and temporoparietal junction (TPJ) (Frith and Frith 1999), while arousal would be revealed by activation changes in insular or subcortical regions. However, it is harder to use neuroimaging to discriminate between models of the audience effect based on self-presentation, on pure mentalising or on communication. All of these processes engage medial prefrontal cortex (Amodio and Frith 2006; Kampe et al. 2003) and are likely to draw on overlapping cognitive representations of the self, the other’s impression of the self and the communicative process needed to change the other. Despite these overlaps, neuroimaging studies can give us a starting point for considering the mechanisms behind audience effects and pointers for future studies with diverse populations.

Few studies have directly examined the audience effect in fMRI, because the scanner environment is not amenable to the type of audience effect studies traditionally implemented in social psychology. However, some relevant papers are available. A study by Somerville et al. (2013) asked participants aged 8–22 years old to complete an fMRI scan while participant’s believed that a camera provided a live feed of the participant’s face to a friend outside the scanner. A simple red light indicated to the participant if the camera was on (audience present) or off (audience absent). When participants believed they could be seen, activation in mPFC increased and mPFC-striatum connectivity was found. This effect peaked in adolescence, which can be linked to the increase in sensitivity to a social audience in this age group (Somerville et al. 2013).

Two studies have examined audience effects in donation tasks. First, participants were given the option to donate to different charities (or keep the money) during fMRI, and were observed on some trials but not others. Ventral striatum, a region associated with reward processing, was engaged on trials where participants decide to donate and could be seen doing so (Izuma et al. 2010a). Second, participants rated how often they engage in prosocial or antisocial behaviours (e.g., *I have said something bad about a friend behind his or her back*), either when being watched by another person or when not being watched. mPFC and dorsal striatum were more strongly engaged when participants were being watched and rated their own behaviours (Izuma et al. 2010b). In both of these studies, the feeling of being watched was generated by showing participants video clips of two observers, and the instruction that these videos were a live feed from an adjacent room where the observers were watching.

A different approach used stories to test the effect of having an audience on social and moral transgressions. Participants in fMRI read short stories in which they committed a social or moral transgression, and were either observed or not observed by other people in the story. Results showed engagement of both medial prefrontal cortex and ventrolateral prefrontal cortex when participants read about all moral transgressions or social transgressions which were observed (Finger 2006). This suggests that being seen to do something socially wrong engages similar processes to moral failings, and again links audience effects to mentalising processes.

Finally, the presence of an audience can easily make people feel embarrassed or self-conscious. A recent study took advantage of this, asking participants in the scanner to make simple estimations (e.g., how heavy is an orange?). Participants believed their responses could be seen by three confederates on some trials but not others, and that their own responses were badly wrong on some trials but not others. Results showed that making public decisions engaged medial prefrontal cortex, and that public failures lead to larger pupil dilations and activation of anterior insula. This links audience effects to both theory of mind and physiological stress responses (Müller-Pinzler et al. 2015).

In addition to these studies which directly examine audience effects, a number of papers have compared neural responses when participants see direct gaze compared to averted gaze, where the former can give the feeling of being watched. Reviewing all these is beyond the scope of the current paper (Hamilton 2016; Senju and Johnson 2009) but the themes remain consistent. Across both gaze papers and audience papers, a common theme is that the sense of an audience is linked to activation in brain areas linked to mentalising and self-related processing, in particular mPFC, and also to reward areas (striatum) as well as other aspects of social cognition. It is worth noting the TPJ, which is particularly important in false-belief tasks (Saxe and Kanwisher 2003), it less commonly engaged than mPFC in the studies reviewed above.

Overall, the studies reviewed above are consistent with the idea that audience effects may be driven by mentalising processes or self-construal processes, rather than only arousal processes. However, the involvement of mPFC is not enough to distinguish between self-presentation theories, reputation management theories and communication theories. All these different processes are known to engage the mPFC, possibly because all rely on aspects of mentalising. In the next section, we move on to consideration of diversity in audience effects and what the mentalising hypothesis can tell us about this. We consider individual differences within the typical population, and developmental populations and atypical social cognition in autism. Reviewing these studies will provide a more complete picture of what the audience effect is and where it comes from.

## Diversity of the participants

### Individual differences within and across cultures

The current paper has so far discussed several features of audience effect paradigms, ranging from the characteristics of the task to different types of audiences. However, it is important to recognise that features of the participants may also affect their response to being watched. Here, we review personality traits (studied within Western populations), cross cultural differences between Western and Eastern cultures, and clinical differences in social anxiety disorder. Examining these different populations can reveal how different people in different contexts may show different reactions to being watched, and thus help us understand the cognitive mechanisms underlying the audience effect.

Numerous studies have investigated personal characteristics such as trait anxiety (Hutchinson and Cotten 1973), self-esteem (Brockner and Hulton 1978) and introversion/extraversion (Grant and Dajee 2003) in relation to the audience effect. An excellent review by Uziel (2007) discusses the role of individual differences in depth and proposes a model to account for different outcomes. He suggests that different personality traits place people on a ‘positive-self-assured’ or a ‘negative-apprehensive’ path when they believe another is observing them. The former group includes those with extraversion and high self-esteem, while the latter group includes those who are introverted and have low self-esteem. In a meta-analysis of 26 studies, Uziel finds that people with positive-self-assured traits respond with better performance when they are observed, while those with negative-apprehensive traits respond worse. In contrast, easy or difficult tasks had less effect on performance, which suggests that personality factors may matter more than task factors in defining the impact of an audience.

Different cultures around the world differ in how much people see themselves as independent of others (typically western cultures) or interdependent with others (typically eastern cultures) (Markus and Kitayama 1991). There is some evidence that this can lead to differences in how people feel about being watched by others too. Kitayama et al. suggest that images of another person (which may imply being watched) invoked anxiety in people from East Asian cultures, who feel they are being monitored for social performance. In contrast, images of another person seem to invoked feelings of safety in American participants. Data supporting this hypothesis come from a study where European/American and Asian participants performed a flanker task following priming by a face or house image. Results showed that Asian participants showed a larger error-related negativity (ERN) following face primes, compared to European/American participants (Park and Kitayama 2014). This can be explained if Asian participants feel more under threat when they are watched, and become more sensitive to their own errors.

In a second study, Hitokoto et al. (2016) measured ERPs in Caucasian and Asian participants performing a gambling task, with priming by a face stimulus before some decisions. As before, Asian participants showed larger ERP signals when receiving feedback after a face prime, compared to American participants. This effect correlated with extraversion and cultural interdependence, such that a larger ERP effect was seen in participants with low extraversion and high interdependence (Hitokoto et al. 2016). This pattern of results is consistent with the idea that the face-primes act as a threat stimulus or a negative audience to participants from interdependent cultures and those with low extraversion.

These cross-cultural results are consistent with Uziel’s claim that personality traits place people on a ‘positive-self-assured’ or a ‘negative-apprehensive’ path when they believe another is observing them. The former group respond positively to being observed, while the latter group respond negatively. If Western participants are more often positive-self-assured and Asian participants are more often negative-apprehensive, then the idea that individual differences between positive-self-assured and negative-apprehensive participants can predict how people respond to being watched, seems to hold both within and between cultures.

A final test of this idea can be found in the study of disorders of social interaction, and how the presence of an audience might affect people with such disorders. For example, social anxiety disorder is an intense fear of evaluation from others, which leads people to avoid social situations (Morrison and Heimberg 2013). It has been described in terms of an intense focus on how other people see you, combined with negative self-image and poor emotion regulation (Morrison and Heimberg 2013; Schlenker and Leary 1982). This could be the extreme of Uziel’s negative-apprehensive path, and suggests that engaging in too much reputation management is harmful. It has further been suggested that the use of positive virtual reality audiences could provide a therapy for people with social anxiety to learn that others evaluate them positively rather than negatively (Pertaub et al. 2002). However, it remains to be seen if social anxiety is more than just an extreme example type of audience effect seen in typical individuals with negative-apprehensive traits.

### Development of audience effects

The vast majority of studies of the audience effect have been conducted in typical adults. However, when considering mentalising, there are a large number of important developmental changes in mentalising abilities, in infancy and childhood (Onishi and Baillargeon 2005; Wellman and Liu 2004) and with developmental changes continuing in adolescence (Dumontheil et al. 2010). These studies suggest that implicit mentalising abilities are present at an early age (approx. 7 months) but performance on some mentalising tasks continues to improve up to age 18. Examining the development of audience effects thus helps us understand the diversity of this phenomena and the links between mentalising and audience effects. Here we review research on the development of the audience effect and then consider how this links to studies of mentalising.

Very few researchers have examined audience effects in infancy. Young children prefer to view faces from birth (Farroni et al. 2002) and 10 month olds produce more smiles when observed (Jones et al. 1991), but other behaviours have not been tested with/without an audience. Studies in older children suggest that audience effects develop gradually. An early study found larger audience effects in 8 year olds compared to 4 year olds performing a balance task (Maccracken and Stadulis 1985). In a dictator game applied to children from 6 to 12 years of age, only children above age 9 were influenced by peer observation of their behaviour (Takagishi et al. 2015). Electrophysiological measures also change with age: ERN recorded from children performing a motor task was larger when an audience was present, but this effect was even larger in older children (9–11 years) compared to younger children (7–8 years). This implies an increase in the audience effect with age. More recent studies focused on social behaviour show that 5 year olds share more and steal less when observed, and that observation by ingroup members has a larger impact (Engelmann et al. 2012, 2013). Leimgruber et al. (2012) reports similar results, with 5 year olds sharing more when observed than when in private (Leimgruber et al. 2012). These studies all imply that reputation management mechanisms develop from 5 years of age. However, few studies have tested younger children or examined the relationship between reputation management and theory of mind, so further study of the early developmental origins of the audience effect would be valuable.

The development of audience effects continues in adolescence, when substantial changes in self-concept, reputation and peer relationships take place. Somerville et al. (2013) discovered a peak in susceptibility for the audience affect during adolescence, compared to during childhood and adulthood. A recent study examined how younger adolescents, older adolescents and adults perform a relational reasoning task in front of either a stranger or a peer (Wolf et al. 2015). Results showed worse performance with a peer audience, particularly in the adolescents. Adolescents also tend to imagine being observed by audiences in everyday life (Sebastian et al. 2008). All these studies show that the propensity to engage in reputation management and consider an audience, especially a peer audience, continues to develop in adolescence.

Overall, these studies demonstrate a protracted developmental trajectory for the audience effect. While even young infants show sensitivity to faces and being observed, full reputation management and the sense of self develop gradually over childhood and adolescence. Future studies could examine how this relates to individual differences such as social anxiety, and the relationship between audience effects and theory of mind across development.

### Audience effects in autism spectrum condition

Individual differences within the typical population and across development clearly impact on audience effects. However, some individual differences require a more in depth approach due to their importance for the understanding of social cognition. Autism spectrum condition (ASC/autism) is a neurodevelopmental disorder with a profound impact on social interaction skills. There are several reasons why autism might lead to different behaviour on audience effect tasks. First, people with autism find mentalising tasks particularly hard (Baron-Cohen et al. 1985; Frith 2012), which has profound effects on their everyday social interactions. Difficulties in metalizing also implies that people with autism should engage less in reputation management and self-enhancement, which leads to the prediction that they should show a reduced audience effect, compared to typical participants. Second, it has recently been suggested that people with autism have a reduced motivation to engage socially (Chevallier et al. 2012), including a reduced desire to maintain a good reputation. This implies that participants with autism have the ability to manage their reputation, but do not want to do so. Finally, around 30 % of people with autism also have social anxiety disorder (Simonoff et al. 2008), which is associated with increased audience effects, especially for negative audiences. Thus, it is interesting to consider if audience effects are enhanced or reduced in autism, and several recent studies have examined this question.

Several studies have examined audience effects in autism in terms of reputation management, and how much people with autism adjust their social behaviour to give a favourable impression to an audience. One way to gain an audience’s favour is by flattery—being complementary to others so they will like you more. Chevallier et al. (2012) found that if typical children (age 12–15 years) are asked to rank pictures, they will give a picture a higher score when they believe the person listening drew the picture, in order to flatter the listener and enhance their own reputation. However, children with autism did not engage in such flattery, suggesting either reduced social motivation or reduced ability to manage ones reputation.

Donation games provide another way to examine reputation management in autism. In a well-controlled study, typical and autistic participants completed blocks of a donation task with or without an observer who noted down responses, supposedly ‘because the computer wasn’t recording data’. Typical participants donated more in the presence of an observer while participants with autism did not. In a control task not involving donations, both typical and autistic participants performed better when being observed (Izuma et al. 2011). This suggests that different mechanisms may govern audience effects in ‘reputation’ tasks versus ‘perceptual’ tasks, with only the former impaired in ASC. In a follow-up study, Cage et al. replicated the finding that typical adults donate more in the presence of an observer while those with autism do not. They further showed that typical adults also donate more when they hope the observer will reciprocate, and that expectation of reciprocity is weaker in ASC. They suggest that people with autism can engage in reputation management (possibly using explicit mentalising strategies) but do not spontaneously do so.

A direct test of the relationship between mentalising and the audience effect is a study by Chevallier et al. (2014), in which children with autism and matched typical children completed a computerised theory of mind test with or without an observer. Typical children showed improved performance in the presence of the observer, but children with ASC showed poor performance in both conditions. Chevallier and colleagues interpret this in terms of a lack of social motivation to perform well in the children with ASC. However, it could also be the case that the ASC group were performing at their best without an observer, and had no extra mentalising capacity to do better when an observer was present. Further studies with a non-social control tasks would be useful to test this hypothesis.

A different approach was taken by Scheeren et al. (2010), who asked control children and children with highly functioning ASD to describe themselves in front of an audience. In one condition they were given information as to what the audience would like to hear, and in the other condition they receive no such information. Both samples spoke more positively about themselves in the former condition compared to the latter, but children with ASD were not as strategic compared to controls in terms of self-promotion. It was argued that this might be because they had difficulty breaking the social and moral rules surrounding lying.

Overall, several studies show a reduction in the audience effect in autism. Two conflicting accounts can be given of this—one possibility is that people with autism struggle with mentalising (Frith 2012) and thus do not appreciate that other people have different beliefs, nor that it is possible to act in a way that changes these beliefs and maintains a good reputation. Second, it is possible that people with autism are not motivated to engage socially with others (Chevallier et al. 2012) and thus do not try to maintain a good reputation. Discriminating between these is not easy on the basis of the current data, and further research in this area will be needed. However, drawing on our previous review of neuroimaging studies of the audience effect, those suggest that brain systems linked to mentalising have a much stronger role in audience effects than those links to motivation, which could favour the mentalising hypothesis.

The studies of audience effects in autism do provide an interesting contrast to the case of social anxiety, reviewed above. People with social anxiety seem to show a large audience effect with intense apprehension about social evaluation (Morrison and Heimberg 2013). In contrast, those with autism show a rather small audience effect and do not engage in reputation management. This suggests that social anxiety may not drive audience effects in those with autism, and raises the interesting question of what type of effect would be found in the 30 % of people with autism who also suffer social anxiety (Simonoff et al. 2008). We are not aware of any studies which have addressed this topic, and it seems an important area to address, which can yield key insights into the nature of social cognition in autism.

## Conclusions

This paper has reviewed a large body of literature on audience effects, over a number of years. It is clear that work on audience effects has moved on from the focus on tasks and arousal that dominated the 60’s. Audience effects are present across a range of tasks including social, economic and emotional tasks in a manner that cannot be explained solely by task difficulty. Rather, it seems that participant characteristics such as introversion/extraversion, anxiety, culture, age and autistic traits are important in determining the type of audience effect observed. This indicates that understanding audience effects may be an important component in understanding the diversity of social cognition.

We have considered here several different theories of the causes of the audience effect, including theories of drive (Zajonc 1965), self-presentation (Bond 1982), communication (Fridlund et al. 1990) and reputation management (Tennie et al. 2010). While it is clear that audience effects involve more than just changes in drive or arousal, it is less easy to distinguish between the other theories. All three draw on the related ideas that, in conditions where an audience effect arises, participants consider their own self-concept, consider how they appear to others, and consider how to communicate a favourable impression to others. While different theories place emphasis on different aspects of this process, it is very hard to separate out the different components, either in behavioural or brain imaging studies. We suggest here that mentalising is a core cognitive component which underlies all the different theories, and that thinking about audience effects in terms of mentalising may give us a step forward in building a cognitive model of this behaviour. However, further studies and theorising will be required to produce a full model.

This review also highlights a number of important future directions for research into audience effects. These include the need to understand individual differences in audience effects, in relation to culture, development, anxiety and autism. The relationship between the reputation management mechanisms engaged in the audience effect, self-related processing and social motivation also need to be clarified, and large studies tracking individual differences in these areas might be helpful. Neuroimaging studies that pinpoint the relationship between motivation, mentalising, gaze and audience effects would also contribute. Overall, the audience effect provides a fine exemplar of a complex social behaviour that draws on many aspects of social skill but can be well controlled in the lab. Unpacking the simple question of ‘what changes when someone watches’, first asked over 110 years ago, may have important implications for our broader models of human social neuroscience and the interactive behaviour across diverse populations.
